# Mercury bioaccumulation by *Suillus bovinus* mushroom and probable dietary intake with the mushroom meal

**DOI:** 10.1007/s11356-016-6558-8

**Published:** 2016-04-12

**Authors:** Martyna Saba, Jerzy Falandysz, Innocent C. Nnorom

**Affiliations:** Laboratory of Environmental Chemistry and Ecotoxicology, Gdańsk University, 63 Wita Stwosza Str., 80-308 Gdańsk, Poland; Environmental Chemistry Unit, Department of Industrial Chemistry, Abia State University, Uturu, Abia State Nigeria

**Keywords:** Forest, Fungi, Heavy metals, *Suillus* mushroom, Organic food, Soil

## Abstract

This paper reports the results of the study of the efficiency of accumulation and distribution of mercury (Hg) in the fruiting bodies of fungus *Suillus bovinus* and the probable dietary intake of Hg and the potential health risk. Fungal fruiting bodies and soil materials were collected from 13 background areas in the northern part of Poland between 1993 and 2013. Mercury in the caps of fruiting bodies varied from 0.10 ± 0.06 to 0.79 ± 0.40 mg kg^−1^ dry biomass and in the stipes from 0.083 ± 0.028 to 0.51 ± 0.22 mg kg^−1^ dry biomass. The mean values of cap to stipe Hg content quotient varied from 1.3 ± 0.2 to 2.6 ± 0.6. The Hg content in the upper 0–10 cm layer of soil substrate varied from 0.015 ± 0.004 to 0.031 ± 0.019 mg kg^−1^ dry biomass. *S. bovinus* could be considered as an efficient accumulator of Hg, at least from low level polluted soils, and the values of Hg bioconcentration factor (BCF) varied from 6.4 ± 2.2 to 45 ± 20 for caps and from 3.8 ± 1.4 to 29 ± 11 for stipes. A conventional meal (300 g) portion of *S. bovinus* foraged from background areas provides Hg dose far below the provisionally tolerable weekly intake or recommended reference dose set for this element by authorities. An examination of published data on Hg in fruiting bodies of fungi genus *Suillus* showed low contamination of specimens foraged from background areas. Also reviewed are published data on Hg in fungi genus *Suillus* collected worldwide.

## Introduction

Mercury along with other elements such as arsenic, lead, and cadmium are important to consider in terms of food chain contamination (McLaughlin et al. 1999). The past two to three decades have witnessed increasing publications evaluating the Hg levels in foods and the environment at state and regional levels. However, the recent publication of the United Nations Environment Programme indicated that this concern is a global issue as these contaminations result more from anthropogenic emissions of Hg (UNEP 2013). The increasing environmental Hg contamination could be attributed to the unique properties of Hg—its low vapor pressure (elemental Hg) and the persistency of the vapors. Studies have reported long-range transportation of Hg at a global scale and deposition of airborne anthropogenic Hg at remote regions (Demers et al. 2007). Elevated amounts of mercury have been observed in mushrooms *Gymnopus erythropus* (Pers.) Antonín, Halling & Noordel. and *Marasmius dryophilus* (Bull.) Murrill which depend on litter as source of food, in remote regions of the Minya Konka (Mt. Gongga) in the Eastern Tibetan Plateau—a site located very far from industrial sources of Hg emissions and other saprophytic species such as *Agaricus arvensis* Schaeff., *Agaricus maleolens* F.H. Møller (current name *Agaricus bernardii* Quél.), and *Coprinus comatus* (O.F. Müll.) Pers. from contaminated urban grounds (Falandysz 2016, Falandysz et al. 2014a, Svoboda and Kalač 2003).

Mercury as a trace element is natural and ubiquitous, in the lithosphere and hydrosphere, with predilection to combine with sulfur (S) and selenium (Se) in the environment, consequent upon which it occurs in foods and feedstuff. Mercury is readily biomethylated into methylmercury, which is then bioaccumulated (usually together with Se for which MeHg is an antagonist in selenocysteine) up the aquatic food chain (Ralston and Raymond 2010). Differences in Hg contents of soils is due to airborne Hg pollution (accumulation in litter and organic layer of soils) or from geogenic Hg (which occurs under the organic horizon layer). Mercury availability to the mycelia, genetic factor, and adaptation to the geochemical composition and anomalies of soil background could be important variables that determine the amounts of Hg observed in mushrooms—as could be observed in several studies (Árvay et al. 2014; Crane et al. 2010; Falandysz 2014; Falandysz and Bielawski 2001, 2007; Falandysz and Drewnowska 2015; Falandysz et al. 2012a, 2012b, 2014b, 2015a; Krasińska and Falandysz 2015, 2016; Kojta et al. 2012, 2015; Wiejak et al. 2014).

Mushrooms foraged from the woodlands and pastures are simple organic foods or food ingredients that are valued worldwide because of their unique taste, fragrance, texture, and contents of basic nutrients (proteins, minerals etc.). Mushrooms are also rather low in fresh product as their moisture content is about 90 % (Falandysz and Borovička 2013). In the modern times some probiotic features (largely antioxidants content etc.) of crude mushrooms have been highlighted (Sarikurkcu et al. 2015). Another particular feature of mushrooms collected in the wild, which can be species-specific, is the abundance of trace elements and minerals, including those toxic to mammals as well as the specific ability of certain species to efficiently accumulate radiocesium (^134/137^Cs) from radioactive fallout (Chojnacka et al. 2012; Falandysz and Brzostowski 2007; Falandysz et al. 1994; 2015b and 2015c; Karadeniz and Yarpak 2010; Tel et al. 2014). Mercury is an element that is known to be hazardous to man in any of its physical and chemical forms, and in mushrooms, inorganic mercury is the dominant form while methylmercury is a minor constituent (Rieder et al. 2011).

Both the mycorrhizal and non-mycorrhizal mushrooms are efficient in mobilizing and subsequently sequestering Hg and other elements from soil/litter substratum into their fruiting bodies (Chudzyński et al. 2009, 2011; Drewnowska et al. 2012, 2014; Falandysz et al. 1996; Mleczek et al. 2015; Nasr and Arp 2011; Nasr et al. 2012). The mushroom mycelia can very efficiently mobilize Hg from mushroom substratum (soil, litter, or wood) and translocate the same to the mushroom fruiting bodies thereby resulting in the observation of elevated amounts of Hg in the morphological parts of the mushroom (the cap and stipe) compared to the Hg levels in the substrate in some cases (Falandysz et al. 2013; Melgar et al. 2009; Tüzen et al. 1998). Studies have shown a very wide variation in the ability (efficiency) of different mushroom species to accumulate Hg (and other heavy metals such as Pb and Cd) (Alonso et al. 2000; Brzostowski et al. 2011; Falandysz et al. 2001a, 2001b, 2003a, 2003b, 2003c, 2003d, 2007; Falandysz and Gucia 2008; Gucia et al. 2012; Vetter and Berta 1997). The observed variations in metal contents of mushrooms as reported in literature have been attributed to several factors by different authors, including geochemical and biochemical factors (e.g., trace element bioavailability in soils, growing period, and age of mycelium). Unfortunately, till date, the roles of these factors and other possible cofounding variable are not very well understood. Mushrooms’ ability to accumulate Hg is readily estimated by calculating the bioconcentration factor (BCF)—which is usually evaluated to understand the bioconcentration potential of the elements by any given species.

The BCF is a quotient of the Hg content of the mushroom fruiting bodies to that of the substrate. Higher BCF indicates accumulation of the elements of the substrate or soils by the mushroom. Studies have reported BCF of more than 1 for many elements in several species of mushroom. A low BCF value (BCF < 1) can indicate low potential of the mushroom species to accumulate a given element or a low bioavailability of the element contained in the substratum whereas a high BCF will show that the metal under consideration is bioaccumulated by the mushroom.

For contaminants that accumulate in the body over time such as lead, cadmium, dioxin, and mercury, the provisional tolerable weekly intake (PTWI) or monthly intake (PTMI) are used as reference values in evaluating the risks of intake of such toxic metals from food consumption. Thus, in evaluating the intake of contaminants that accumulate in the body over time (such as Hg) from food and foodstuff, the PTWI or PTMI of Hg is usually used as a reference in estimating and evaluating possible risks from intakes of contaminants from food consumption. The Hg PTWI value which was 0.005 mg Hg kg^−1^ bm between 1978 and 2010 was reviewed downwards to 0.004 mg Hg kg^−1^ bm in 2010 based on the assumption that the predominant form of Hg in foods, other than fish and shellfish, is inorganic Hg (JECFA 2010). Similarly, in estimating non-carcinogenic health effects of Hg, a reference dose (RfD) of 0.0003 mg Hg kg^−1^ bm daily is commonly used (US EPA 1987).

This paper reports the results of the investigation of the extent of Hg contamination and bioconcentration of *Suillus bovinus* mushroom collected from forested areas of Poland over a period of about two decades. This study also estimated Hg intake and evaluated the potential human health risk from the consumption of *S. bovinus* collected from within the localities studied thereby providing information necessary in evaluating the likely toxicological implications of the consumption of *S. bovinus* mushroom. Also presented is a review and comparative analysis of Hg in *Suillus* mushrooms.

## Materials and methods

### Sample collection and preservation

**Fig. 1:**
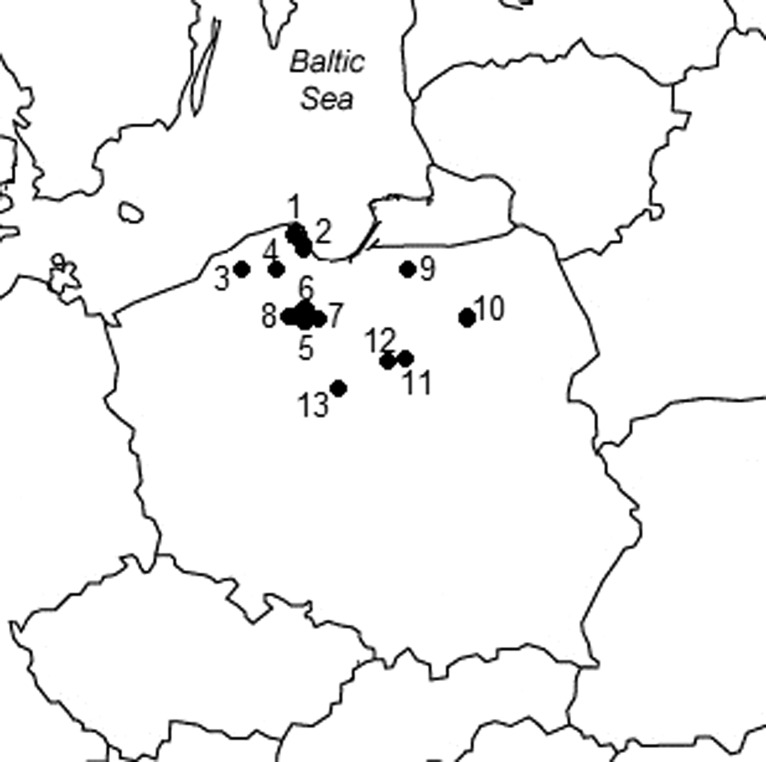
Localization of the sampling places of *S. bovinus* in Poland (*1*–*13*; for details, see Table 1) (*1* Darżlubska Wilderness, *2* Nearshore Landscape Park, *3* Studnica River Valley, Kępice, *4* Sulęczyno, *5* Tuchola Pinewoods, Łuby, *6* Tuchola Pinewoods, Osowo Leśne, *7* Tuchola Pinewoods, Lubichowo, *8* Tuchola Pinewoods, *9* Kaszuny, *10* Szczytno, *11* Kościelna Wieczfnia, *12* Lipowiec Kościelny, *13* Ciechocinek)

During the mushroom collection season in 1993–2013, 586 individual fruiting bodies of *S. bovinus* mushroom were collected from 13 spatially distant places in Poland (Fig. 1). On collecting the mushroom fruiting bodies, the topsoil layer of the forests (0–10 cm) beneath the fruiting bodies were also collected for most of the places studied. The samples were collected from such places in Poland as the Darżlubska Wilderness in Krokowa region, Nearshore Landscape Park, the Studnia river Valley in the region of Kępice, outskirts of the Sulęczyno place in Kaszuby land, from several regions of the Tuchola Pinewoods complex and the forests in the outskirt of Kaszuny and Szczytno in the Warmia and Mazurian lands, Lipowiec Kościelny and Kościelna Wieczfnia in Mazovia land, and Ciechocinek in Kujawy land (Fig. 1, Table 1).

**Table 1:** Mercury in fruiting bodies of European Cow Bolete *Suillus bovinus* (mg kg^−1^ db) and values of the quotients Hg_C_/Hg_S_ Q_C/S_, BCF (arithmetic mean, standard deviation, median, and range)

Place, year, and sample size	Mercury content (mg kg^−1^ dry matter)			*Q*_C/S_	BCF	
	Cap	Stipe	Soil		Cap	Stipe
(1) ^a^Pomerania land, Darżlubska Wilderness, 2003, *n =* 15^b^	0.79 ± 0.4	0.51 ± 0.22	0.017 ± 0.003	1.7 ± 1.1	45 ± 20	29 ± 11
	0.30–1.7	0.14–0.88	0.011–0.021	1.0–5.3	19–96	7.2–49
	0.66	0.43	0.019	1.4	40	28
(2) Pomerania land, Nearshore Landscape Park, 2006, *n =* 15^c^(79)^d^	0.10 ± 0.06	0.083 ± 0.028	0.016 ± 0.007	1.3 ± 0.6	8.1 ± 4.9	5.9 ± 1.9
	0.015–0.26	0.048–0.14	0.0083–0.035	0.17–2.5	1.2–17	1.9–9.5
	0.11	0.082	0.013	1.4	8.4	6.1
(3) Pomerania land, Studnica river Valley, Kępice, 2003, *n =* 14(106)	0.41 ± 0.30	0.20 ± 0.14	0.025 ± 0.011	2.2 ± 0.8	19 ± 16	9.4 ± 7.5
	0.098–1.3	0.041–0.59	0.0069–0.048	0.67–4.0	2.4–64	1.1–30
	0.37	0.17	0.023	2.1	16	7.7
(4) Pomerania land, Sulęczyno 2006, *n =* 15(48)	0.15 ± 0.05	0.091 ± 0.03	0.025 ± 0.006	1.8 ± 0.8	6.4 ± 2.2	3.8 ± 1.4
	0.10–0.31	0.039–0.16	0.015–0.033	0.96–3.5	4.2–10	1.5–6.2
	0.13	0.095	0.026	1.5	5.8	3.6
(5) Pomerania land, Tuchola Pinewoods, Łuby 1993, *n =* 1(25)	0.23		NA	NA	NA	
(6) Pomerania land, Tuchola Pinewoods, Osowo Leśne 2006, *n =* 15(68)	0.21 ± 0.03		0.015 ± 0.004	NA	14 ± 2	
	0.13–0.25		0.0095–0.021		10–19	
	0.22		0.015		14	
(7) Pomerania land, Tuchola Pinewoods, Lubichowo, 2007, *n =* 13(27)	0.28 ± 0.11	0.17 ± 0.07	0.021 ± 0.016	1.8 ± 0.6	18 ± 10	11 ± 7
	0.16–0.60	0.063–0.29	0.0090–0.065	1.1–3.5	4.2–38	2.7–23
	0.26	0.15	0.015	1.6	19	10
(8) Pomerania land, Tuchola Pinewoods, 2011, *n =* 1(60)	0.17		NA	NA	NA	NA
(8) Pomerania, Tuchola Pinewoods, 2013, *n =* 1(62)	0.15		NA	NA	NA	NA
(9) Warmia and Mazury land, Kaszuny, 2003, *n =* 14	0.33 ± 0.19	0.16 ± 0.10	0.025 ± 0.003	2.0 ± 0.7	13 ± 8	6.6 ± 4.2
	0.20–0.78	0.070–0.49	0.018–0.031	1.4–4.2	4.2–3.1	3.0–19
	0.28	0.15	0.025	1.9	11	5.5
(10) Warmia and Mazury land, Szczytno, 2003, *n =* 14	0.20 ± 0.04	0.10 ± 0.04	0.031 ± 0.019	2.2 ± 0.6	8.6 ± 3.8	4.4 ± 1.9
	0.13–0.26	0.043–0.15	0.012–0.073	1.4–3.5	3.3–14	1.3–7.9
	0.22	0.088	0.021	2.0	9.3	4.7
(11) Mazovia land, Commune of Kościelna Wieczfnia, 2006, *n =* 14(15)	0.11 ± 0.03	0.083 ± 0.030	0.017 ± 0.014	1.3 ± 0.2	9.4 ± 5.6	7.3 ± 4.7
	0.056–0.18	0.055–0.17	0.006–0.62	0.95–1.8	1.4–20	1.0–15
	0.11	0.080	0.013	1.3	7.1	5.2
(12) Mazovia land, Lipowiec Kościelny, 2006, *n =* 15(20)	0.38 ± 0.18	0.16 ± 0.13	0.027 ± 0.012	2.6 ± 0.6	19 ± 14	7.4 ± 5.4
	0.19–0.99	0.07–0.61	0.01–0.04	1.6–4.0	8.0–50	2.4–19
	0.37	0.15	0.03	2.5	12	5.1
(13) Kujawy land, Ciechocinek, 2004, *n =* 15(33)	0.26 ± 0.06	0.18 ± 0.04	0.026 ± 0.004	1.5 ± 0.3	10 ± 3	6.8 ± 2.0
	0.20–0.38	0.12–0.25	0.018–0.032	1.1–2.2	6.7–16	4.1–10
	0.24	0.17	0.026	1.5	9.2	6.6

^a^Place (see Fig. 1)

^b^Number of individuals

^c^Number of composite samples

^d^Number of individuals in a pool (in parentheses)

All individual fruiting bodies selected for this study were mature and in good body condition (not infected by insects). The mushroom fresh fruiting bodies were cleaned up from any visible plant vegetation and soil debris with a plastic knife. To get insight into the distribution of Hg between the two major morphological parts of the fruiting bodies of mushrooms, the individual mushrooms from several places were separated into cap (with skin) and stipe. Next, the individual cap and stipe samples were sliced using a plastic knife and dried separately or in a pool accordingly (Falandysz 2014). Thereafter, for drying, the mushroom samples were placed into a plastic basket of the electrically heated commercial dryer for vegetables and dried at 65 °C to constant mass. Dried fungal materials were pulverized in a porcelain mortar and kept in brand new sealed polyethylene bags under dry conditions. The soil samples, free of any visible organisms, small stones, sticks, and leaves were air dried at room temperature for several days under clean conditions and further dried at 65 °C to constant mass. Next, the soil samples were ground in a porcelain mortar, sieved through a pore size of 2-mm plastic sieve, and thereafter stored in brand new sealed polyethylene bags under dry conditions.

Double distilled water was used in all preparations. Mercury standard solution of 1.0 mg mL^−1^ was obtained from the 10 mg mL^−1^ standard stock solution. Blank and 100, 150, and 200 μL of 1.0 mg mL^−1^ Hg standard solutions were injected into the analyzer for the construction of a calibration curve, which was prepared new each week.

### Sample analyses

The determinations of total Hg content of fungal and soil samples was performed using cold vapor atomic absorption spectroscopy (CV-AAS) by a direct sample thermal decomposition coupled with gold wool trap of Hg and its further desorption and quantitative measurement at wavelength of 296 nm. The analytical instrument used was mercury analyzer (MA-2000, Nippon Instruments Corporation, Takatsuki, Japan) equipped with auto sampler and operated respectively at low and high modes (Jarzyńska and Falandysz 2011; Nnorom et al. 2013).

A running analytical control and assurance quality (AC/AQ) was performed through the analysis of blank samples and certified fungal reference materials produced by the Institute of Nuclear Chemistry and Technology, Warsaw, Poland. The declared content of Hg for material CS-M-1 (dried mushroom powder *S. bovinus*) is 0.174 ± 0.018 Hg mg kg^−1^ db Hg, and our result (*n* = 13) was 0.185 ± 0.011 mg kg^−1^ db; for CS-M-2 (dried mushroom powder *Agaricus campestris*), the declared Hg content is 0.164 ± 0.004 Hg mg kg^−1^ db and our result (*n* = 8) was 0.165 ± 0.005 mg kg^−1^ db. The limit of detection (LOD) of this study was 0.003 mg Hg kg^−1^ dm, and the quantification limit (LOQ) was 0.005 mg Hg kg^−1^ db. One blank sample and one certified reference material sample were examined with each set of three to five samples studied.

### Target hazard quotient (THQ)

The estimated daily intake (EDI) was calculated to estimate the potential hazard from the consumption of a meal of *S. bovinus* using the formula:

$$ \mathrm{E}\mathrm{D}\mathrm{I}=\frac{\mathrm{Mc}\times \mathrm{Consumption}\ \mathrm{rate}}{\mathrm{Body}\;\mathrm{weight}} $$

where Mc is the Hg content of the mushroom (mg kg^−1^, fresh weight), consumption rate of 100 g day^−1^ and 300 g day^−1^ for average level consumer (ALC) and a high level consumer (HLC), respectively, while the body weight for ALC was 30 kg and that of HLC is 70 kg.

Similarly, to assess the long-term potential health risks associated with Hg intakes from consumption of *S. bovinus*, the target hazard quotients (THQ) was calculated to evaluate the non-carcinogenic health risk. The THQ is often used in evaluating potential risks for Hg (as well as other heavy metals) intakes from contaminated foods. THQ was proposed by the USEPA (USEPA 2000). THQ values greater than 1 indicates that the consumption of contaminated foods is likely to expose the consumer to risks that could result in deleterious effects. In this study, non-cancer risk assessment of Hg exposure from consumption of *S. bovinus* was evaluated based on the use of THQ which is a ratio between the estimated dose of contaminant Hg and the reference dose below which there will not be any appreciable risk. The method used for the evaluation of THQ is described by the equation below (Chien et al. 2002; US EPA 2000; Wang et al. 2005).

$$ \mathrm{T}\mathrm{H}\mathrm{Q}=\frac{E_{\mathrm{F}}{E}_{\mathrm{D}}{F}_{\mathrm{IR}}C}{{\mathrm{RfD}}_{\mathrm{o}}\;{W}_{\mathrm{A}\mathrm{B}}\;{T}_{\mathrm{A}}}\times {10}^{-3} $$

*E*_F_ is the exposure frequency (365 days year^−1^); *E*_D_ is the exposure duration (70 years), equivalent to the average lifetime (Bennett et al. 1999); *F*_IR_ is the mushroom ingestion rate (g person day^−1^), assuming 300 g fresh mushroom consumption for HLC (adults) and 100 g for ALC (children); *C* is the average Hg content in mushroom (mg kg^−1^ wet weight); RfDo is the mercury oral reference dose (0.0005 mg kg^−1^ day^−1^) (US EPA 1997); *W*_AB_ is the average body weight (70 kg for a HLC and 30 kg for ALC), and *T*_A_ is the averaged exposure time for non-carcinogens (365 days year^−1^ × *E*_D,_ assuming 70 years for HLC and 30 years for ALC). An index more than 1 is considered as not safe for human health (USEPA 2002), and this indicates that it is likely that the consumer of the contaminated food may experience deleterious effects. The higher the THQ, the higher the chances of risk to the exposed population.

## Results and discussion

### Hg in mushroom cap and stipe

The mercury contents of the caps and stipes of *S. bovinus* mushroom and the soils beneath fruiting body as well as the values of the quotients of Hg content of cap to stipe (*Q*_C/S_) and the quotient of Hg in cap/stipe to Hg in soil substratum (BCF; bioconcentration factor) are given in Table 1 (on a dry biomass basis, db). The mercury contents of the soil samples and fruiting bodies (cap and stipe or the whole fruiting bodies) of the European Cow Bolete *S. bovinus* samples were all found to be less than 1.0 mg kg^−1^ db.

The mean Hg concentrations of the fruiting bodies of *Suillus bovines* revealed slightly elevated Hg uptake for samples from Pomerania land and Darżlubska Wilderness vicinity compared to the other sites. The mean Hg in the cap varied from 0.10 ± 0.06 mg kg^−1^ db (Pomerania, Nearshore landscape Park) to 0.79 ± 0.4 mg kg^−1^ db (Pomerania Land, Darżlubska Wilderness site) with Hg in individual samples ranging from 0.015 to 1.7 mg kg^−1^ db. Similar mean Hg in caps values were observed for the Pomerania, Nearshore Landscape Park and Mazovia land, commune of Kościelna Wieczfnia sites (0.10 ± 0.06 and 0.11 ± 0.03 mg kg^−1^ db, respectively), as well as in the caps from Warmia and Mazury land, Szczytno (0.20 ± 0.04 mg kg^−1^ db) and in whole fruiting bodies from Pomerania land, Tuchola Pinewoods, Osowo Leśne (0.21 ± 0.03 mg kg^−1^ db). The median mercury concentration of the cap of *S. bovinus* samples ranged from 0.11 to 0.66 mg kg^−1^ db. In this work, higher Hg contents were observed in cap compared to the stipes, with Qc/s values ranging from 1.3 ± 0.2 to 2.6 ± 0.6 with individual Qc/s values ranging from 0.17 to as high as 5.3.

### Hg in soils

The mean Hg in soil samples ranged from 0.015 ± 0.004 mg kg^−1^ db (Pomerania land, Tuchola Pinewoods, Osowo Leśne) to 0.031 ± 0.019 mg kg^−1^ db in Warmia and Mazury land, Szczytno). Similar Hg in soil values were observed for the Pomerania land Studnica river valley, the Kępice and Sulęczyno as well as the Warmia and Mazury land Kaszuny sites (0.025 ± 0.011, 0.025 ± 0.006, and 0.025 ± 0.003 mg kg^−1^ db respectively), as well as for the Pomerania land, Darżlubska Wilderness vicinity and Mazovia land, commune of Kościelna Wieczfnia sites (0.017 ± 0.003 and 0.017 ± 0.014 mg kg^−1^ db respectively). The Hg content in the soil samples ranged from 0.0069 to 0.62 mg kg^−1^ db (median values varied from 0.013 to 0.26 mg kg^−1^ db).

### Bioconcentration potential

To assess the potential of mushroom to take-up and sequester elements (Hg in this case) in fruiting body, the quotient of Hg in the mushroom fruiting bodies (cap or stipe or even the whole fruiting body) to the corresponding Hg in litter/soil layer of the substratum which is defined as BCF or transfer factor (TF) is readily used. In this study, the BCF values ranged from 6.4 ± 2.2 to 45 ± 20 (individual BCF values ranging from 1.2 to 96) for caps and from 3.8 ± 1.4 to 29 ± 11 (individual BCF values ranging from 1.1 to 49) for stipes indicating that *S. bovinus* is a moderate accumulator of mercury (Table 1).

For the range of rather low Hg concentrations determined in the forest topsoil layer samples in this study, no statistically significant relationship could be observed between Hg level in soil substrate and Hg accumulated in fruiting bodies of *S. bovinus*. A positive tendency of an increase in the Hg content of mushrooms with increase in Hg levels in soils was observed for a set of mushrooms of genus *Leccinum* that emerged from soils with a wide range of Hg content—from low to elevated soil Hg contents, probably resulting from geogenic anomaly (Falandysz et al. 2015a).

### Hg intake from consumption of *S. bovinus*

For health concerns, especially considering the dangers of intakes of toxic contaminants such as mercury, it is pertinent to estimate Hg intakes from food and foodstuff. This allows the evaluation of either the nutritional benefits or toxicological concerns of intakes from consumption of such foods. A mercury RfD of 0.0003 mg kg^−1^ body mass daily which was set by the US EPA is readily used when evaluating the risks in Hg intake from foods. Also used in such assessments is the tolerable daily intake rate of 0.00061 mg kg^−1^ body mass which is derived from the PTWI of 0.0043 mg kg^−1^ body mass (i.e., 0.26 mg for an individual of 60 kg body mass) as established by the FAO/WHO (US EPA 1987; JECFA 2010). Literature identifies mushroom meals to be in the range of 100–500 g per meal depending on variables such as mushroom availability (during or out of the mushrooming season) and individual preference. In this study, intake rates of Hg from consumption of *S. bovinus* was estimated by assuming that an average consumer eats about 300 g of caps of *S. bovinus* at the site surveyed. The consumption of a meal 300 g of *S. bovinus* collected at the Mazovia land commune of Kościelna Wieczfnia (site with the lowest mean Hg in caps of 0.11 ± 0.03 mg kg^-1^ db) will result in estimated Hg intake of 0.0033 mg while for the Pomerania land, Darżlubska Wilderness site (site with the highest mean Hg in cap of 0.79 ± 0.40 mg kg^−1^ db), this will result in estimated Hg intake of 0.024 mg. These estimated intake values were calculated assuming 90 % moisture content of mushroom and an adult individual of 60 kg body mass.

These exposure levels will amount to 1.4 to 10 % of the recommended reference dose for caps on consumption of 300 g of caps only and these are below the 0.24 mg Hg dose of PTWI, assuming no Hg from other foods is ingested. Consequently, there is no toxicological concern over Hg intake from the consumption of *S. bovinus* from the locations investigated in this study.

### Target hazard quotient (THQ)

The EDI and THQ values on the consumption of *S. bovinus* by ALC who consumes a 100 g meal of caps of *S. bovinus* (fresh weight) and a HLC who consumes 300 g of this mushroom for the sites studied (using the range of the data obtained) are presented in Table 2.

**Table 2:** Values of estimated daily intakes (EDI) (μg/kg fresh weight/day) and target hazard quotient (THQ) of an average level consumer (ALC) and a high level consumer (HLC) of *Suillus bovinus* mushroom in Poland

Site	EDI ALC	EDI HLC	THQ ALC	THQ HLC
Pomerania land, Darżlubska Wilderness	0.0003^a^–0.0005^b^	0.000128–0.000728	0.00019–0.00113	0.000257–0.001457
Pomerania land, Nearshore Landscape Park	0.000005–0.000086	0.000006–0.000011	0.000009–0.000173	0.000013–0.000223
Pomerania land, Studnica river Valley, Kępice	0.000001–0.000043	0.000042–0.000055	0.000065–0.000866	0.000083–0.001114
Pomerania land, Sulęczyno	0.000003–0.000010	0.000004–0.000013	0.000066–0.000206	0.000085–0.000265
Pomerania land, Tuchola Pinewoods, Osowo Leśne	0.000004–0.000008	0.000005–0.000010	0.000086–0.000166	0.000111–0.000214
Pomerania land, Tuchola Pinewoods, Lubichowo	0.000005–0.000200	0.000006–0.000025	0.000106–0.000399	0.000137–0.000514
Warmia and Mazury land, Kaszuny	0.000006–0.000260	0.000008–0.000033	0.000133–0.000519	0.000171–0.000668
Warmia and Mazury land, Szczytno	0.000004–0.000008	0.000005–0.000011	0.000086–0.000173	0.000111–0.000223
Mazovia land, Commune of Kościelna Wieczfnia,	0.000018–0.000060	0.000024–0.000077	0.000037–0.000119	0.000047–0.000154
Mazovia land, Lipowiec Kościelny	0.000063–0.000330	0.000081–0.000420	0.000126–0.000659	0.000163–0.000848
Kujawy land, Ciechocinek	0.000006–0.000126	0.000085–0.000016	0.000133–0.000253	0.000171–0.000325

^a^Minimum

^b^Maximum

Values of EDI (μg kg^−1^ fresh weight/day) and THQ for all the sites were generally very low for both ALC and HLC indicating that the consumption of *S. bovinus* does not pose health hazard to consumers. THQ is gaining prominence as one of the parameters used in assessing risk of exposure from food consumption. The THQ values were <1 for all sites studied, including the Pomerania land, Puszcza Darżlubska site for which the highest Hg in caps (1.7 mg kg^−1^) of this study was observed (Table 2). This indicates that the consumers of *S. bovinus* for the sites investigated are not exposed to hazard risks.

### Review of literature on Hg in fruiting bodies of fungi genus *Suillus*

Available literature of Hg contents for 12 species of fungi genus *Suillus* are given in Table 3. Data presented in Table 3 include species such as *S. bovinus* (L.) Roussel, *Suillus brevipes* (Peck) Kuntze, *Suillus cavipes* (Opat.) A.H. Sm. & Thiers, *Suillus collinitus* (Fr.) Kuntze, *Suillus granulatus* (L.) Roussel (also called *Suillus lactifluus* With.), *Suillus grevillei* (Klotzsch) Singer, *Suillus luteus* (L.) Roussel, *Suillus placidus* (Bonord.) Singer, *Suillus spraguei* (Berk. & M.A. Curtis) Kuntze (formally called *Suillus pictus* Kuntze), *Suillus variegatus* (Sw.) Richon & Roze, and *Suillus viscidus* (L.) Roussel (formally called *Suillus aeruginascens* (Species Fungorum 2015). In several studies with varying numbers of specimens and from diverse sampling sites/locations, the typical content of Hg in *Suillus* mushrooms as reviewed (Table 3) was <1 mg kg^−1^ db for whole fruiting bodies, <1 mg kg^−1^ db for caps, and <0.5 mg kg^−1^ db for stipes. When compared to the “typical content,” a substantially elevated Hg content have been observed in individual samples of three species collected from sites polluted with Hg. Due to the operation of a mercury smelter and a copper smelter in the Middle Spiš land in the Slovakia (Central Europe), high Hg levels were reported in *Suillus* species from this site—5.7 mg kg^−1^ db in *Suillus granulatus*, 4.8 mg kg^−1^ db in *Suillus grevillei*, and 5.8 mg kg^−1^ db in *Suillus luteus* (Table 3). An elevated Hg content of 2.5 mg kg^−1^ db (which is above the “typical level”) was also reported for *S. granulatus* from a location with no known history of Hg pollution (substrate is the possible source) in Hungary. For majority of the *Suillus* mushrooms reviewed and with relatively large datasets, regardless of the origin/source of the specimens (mostly European in this review), the Hg content in fruiting body and its morphological parts was well below 0.5 mg kg^−1^ db. Data available on Hg in *Suillus* mushrooms and in their soil substrate polluted (from a point source or from global depositions) with Hg could imply that they are able to respond in a dose-response manner to elevated exposure (via soil substrata) to Hg regardless of the origin of the Hg—geogenic (natural) or anthropogenic (non-ferrous smelters). The decades of global Hg emissions from anthropogenic sources and subsequent pollution of forest topsoil with Hg seem to have had little effect on the levels of Hg accumulated in fruiting bodies by mycorrhizal fungi of genus *Suillus*. Nevertheless, no data are available on Hg contents of mushrooms from the pre-industrial era.

**Table 3:** Mercury in mushrooms of the genus *Suillus* worldwide (mean; mean ± S.D.; range of the mean values and the overall range—in parentheses, respectively; mg kg^−1^ db), data adapted to show only two significant figures—where necessary

Species, year(s), and number of specimens	Region of the world	Hg (whole fruit body)	Hg (caps)	Hg (stipes)	Reference
*Suillus bovinus*, 1979–80 (*1*)	Japan, Fukushima	0.6			Kawai et al. 1986
*Suillus bovinus*, p.1976 (*1*)	Europe, Slovenia, Kurešček	0.14			Byrne et al. 1976
*Suillus bovinus*, 1994 (*15*)	Europe, Poland, Kaszuby		0.065 ± 0.026 (0.017–0.13)	0.045 ± 0.023 (0.016–0.10)	Falandysz et al. 1996
*Suillus bovinus*, 1995–96 (*15*)	Europe, Poland, Kaszuby		0.32 ± 0.17 (0.18–0.75)	0.16 ± 0.07 (0.040–0.29)	Falandysz et al. 2003b
*Suillus bovinus*, 1996–97 (*14*)	Europe, Poland, Pomerania		0.65 ± 0.50 (0.14–1.8)	0.35 ± 0.18 (0.10–0.65)	Falandysz et al. 2003a
*Suillus bovinus*, 1997–98 (*11*)	Europe, Poland, Pomerania		0.20 ± 0.11 (0.090–0.41)	0.077 ± 0.035 (0.038–0.15)	Falandysz et al. 2004
*Suillus bovinus*, 1994 (*3*)	Europe, Poland, Pomerania		0.26 ± 0.02 (0.25–0.28)		Falandysz et al. 2001b
*Suillus bovinus*, (*1*) ^a^	Europe, Poland	0.17 ± 0.02			Falandysz et al. 2015c
*Suillus bovinus*, 2002 (*1*)	Asia Minor, Turkey, Izmir	0.10 ± 0.01			Kardeniz and Yaprak 2010
*Suillus bovinus*, 2002 (*1*)	Asia Minor, Turkey, Kurudere	0.050 ± 0.005			Karadeniz and Yarpak 2010
*Suillus bovinus*, 1993–2013 (*586*)	Europe, Poland	0.15-0.23	0.10 ± 0.06–0.79 ± 0.40	0.083 ± 0.028–0.51 ± 0.22	This study
*Suillus brevipes*, p.2006 (*1*)	China, Sichuan, Liangshan	0.12			Zhang et al. 2006
*Suillus cavipes*, p.2012 (*22*)	N. America, New Brunswick	0.20 ± 0.10 (0.10–5.1)			Nasr et al. 2012
*Suillus collinitus*, p.2006 (*14*)	Europe, Italy, R. Emilia	0.18			Cocchi et al. 2006
*Suillus collinitus*, 2013–14 (*52*)	China, Yunnan, Yuxi		0.089–0.42	0.040–0.20	Cocchi et al. 2006
*Suillus granulatus*, 1967/1974 (*5*)	Europe, Germany - south	0.37 (0.25–0.65)			Seeger and Nützel 1976
*Suillus granulatus*, 1990–99 (*2*)	Europe, Slovakia, Middle Spiš	5.7			Zimmermannová et al. 2001
*Suillus granulatus,* 1993–94 (*15*)	Europe, Poland, Kaszuby		0.18 ± 0.08 (0.055–0.33)	0.070 ± 0.029 (0.037–0.14)	Falandysz et al. 1996
*Suillus granulatus*, 2005 (*1*)	Europe, Poland, Łódź	0.24	0.32	0.16	Szynkowska et al. 2008
*Suillus granulatus*, p.2006 (*1*)	China, Sichuan, Liangshan	0.28			Zhang et al. 2006
*Suillus granulatus*, p.2006 (*17*)	Europe, Italy, R. Emilia	0.29			Cocchi et al. 2006
*Suillus granulatus*, 1993 (*1*)	Europe, Hungary	2.5 ± 0.0			Vetter and Berta 1997
*Suillus granulatus*, 2002 (*52*)	Europe, Poland		0.38–0.41	0.068–0.14	Saba et al. 2016b
*Suillus granulatus*, 2003 (*1*)	Europe, Sweden, Forsmark	0.069			Johanson et al. 2004
*Suillus grevillei*, 1967–74 (7)	Europe, Germany - south	0.22 (0.08-0.45)			Seeger and Nützel, 1976
*Suillus grevillei*, 1990–99 (*4*)	Europe, Slovakia, Middle Spiš	4.8			Zimmermannová et al. 2001
*Suillus grevillei*, 1997–98 (*15*)	Europe, Poland, Pomerania		0.22 ± 0.06 (0.080–0.32)	0.13 ± 0.05 (0.065–0.24)	Falandysz et al. 2004
*Suillus grevillei*, 2000–2006 (*121*)	Europe, Poland		0.26 ± 0.08–0.50 ± 0.10	0.089 ± 0.026–0.16 ± 0.07	Chudzyński et al. 2009
*Suillus grevillei*, 1993 (*1*)	Europe, Hungary	0.10 ± 0.00			Vetter and Berta 1997
*Suillus grevillei*, 2000 (*1*)	Asia, Katun Nature Resrve		0.1	0.1	Gorbunova et al. 2009
*Suillus grevillei*, p.2011 (*1*)	Europe, Switzerland	0.38			Rieder at al. 2011
*Suillus grevillei*, p.2012 (*40*)	N. America, New Brunswick	0.56 ± 0.80 (0.30–2.9)			Nasr et al. 2012
*Suillus luteus*, 1967 (*1*)	Europe, Germany—south	0.15			Seeger and Nützel 1976
*Suillus luteus*, 1990–99 (*10*)	Europe, Slovakia, Middle Spiš	5.8			Zimmermannová et al. 2001
*Suillus luteus*, 1997–98 (*15*)	Europe, Poland, Pomerania		0.13 ± 0.06 (0.061–0.23)	0.054 ± 0.037 (0.017–0.16)	Falandysz et al. 2004
*Suillus luteus*, 1995–96 (*15*)	Europe, Poland, Kaszuby		0.19 ± 0.07 (0.12–0.39)	0.088 ± 0.042 (0.041–0.13)	Falandysz et al. 2003b
*Suillus luteus*, p.2006 (*17*)	Europe, Italy	0.28			Cocchi et al. 2006
*Suillus luteus*, 1995 (*14*)	Europe, Sweden		0.17 ± 0.07	0.074 ± 0.014	Saba et al. 2016a
*Suillus luteus*, 2012–13 (*30*)	Europe, Belarus		0.090–0.15	0.038 0.073	Saba et al. 2016a
*Suillus luteus*, 1997–98 (*96*)	Europe, Poland, Mazury		0.14 ± 0.03 (0.080–0.18)	0.043 ± 0.015 (0.019–0.079)	Falandysz et al. 2002
*Suillus luteus*, 1994 (*3*)	Europe, Poland, Pomerania		0.34 ± 0.05 (0.29–0.38)		Falandysz et al. 2001b
*Suillus luteus*, 2002–07 (*383*)	Europe, Poland		0.095 ± 0.082–0.28 ± 0.07	0.045 ± 0.018–0.13 ± 0.03	Chudzyński et al. 2011
*Suillus luteus*, 2000–2010 (*821*)	Europe, Poland	0.11–0.86 (*342*)	0.13–0.33	0.038–0.096	Saba et al. 2016a
*Suillus luteus*, 1995–2006 (*529*)	Europe, Poland	0.11–0.30	0.29 (*15*)		Saba et al. 2016a
*Suillus luteus*, p.2011 (*1*)	Europe, Switzerland	0.51			Rieder at al. 2011
*Suillus placidus* 1967 (*1*)	Europe, Germany - south	0.12			Seeger and Nützel 1976
*Suillus spraguei*, 2014 (*16*)	China, Yunnan, Pu’er		0.42	0.14	Falandysz et al. 2016
*Suillus spraguei*, 2014 (*19*)	China, Yunnan, Yuxi		0.22	0.10	Falandysz et al. 2016
*Suillus variegatus*, 1967/1974 (*4*)	Europe, Germany - south	0.22 (0.16–0.30)			Seeger and Nützel, 1976
*Suillus variegatus*, 1995–96 (*14*)	Europe, Poland, Kaszuby		0.065 ± 0.033 (0.019–0.12)	0.029 ± 0.014 (0.013–0.060)	Falandysz et al. 2003b
*Suillus variegatus*, 1997–98 (*48*)	Europe, Poland, Mazury		0.26 ± 0.08 (0.12–0.43)	0.084 ± 0.029 (0.050–0.16)	Falandysz et al. 2002
*Suillus variegatus*, 1977–1999 (*10*)	Europe, Finland	0.24			Pelkonen et al. 2008
*Suillus variegatus*, p.2004	Europe, Bohemia	0.24 ± 0.04			Řanda and Kučera 2004
*Suillus variegatus*, 2003 (*3*)	Europe, Sweden, Forsmark	0.14–2.0			Johanson et al. 2004
*Suillus variegatus*, 1998–2013 (*198*)	Europe, Poland	0.087 (*15*)	0.094 ± 0.018–0.27 ± 0.10	0.045 ± 0.08–0.18 ± 0.12	Saba et al. 2016b
*Suillus viscidus*, 1967/1974 (*3*)	Europe, Germany - south	0.27 (0.20–0.35)			Seeger and Nützel, 1976
*Suillus viscidus*, 1996	Europe, East Bohemian	0.80 ± 0.21			Cibulka et al. 1996
*Suillus viscidus*, 1996	Europe, West Bohemian	1.0 ± 0.6			Cibulka et al. 1996
*Suillus viscidus*, 1996	Europe, North Bohemian	0.76 ± 0.26			Cibulka et al. 1996
*Suillus viscidus*, p.2012 (*12*)	N. America, New Brunswick	0.33 ± 0.19 (0.15-0.77)			Nasr et al. 2012

^a^CS-M-1 (dried fruiting bodies of mushroom Cow Bolete *Suillus bovinus*), produced by the Institute of Nuclear Chemistry and Technology in Warsaw, Poland); unpublished (own study)

## Conclusion

This study has shown that *S. bovinus* collected from sites considered as non-contaminated with Hg in Poland contain low Hg levels that varied spatially in both the caps and the stipes. The low Hg contents of the substrate suggest some airborne Hg deposition and low geogenic contamination with mercury and that for less contaminated soils which can be found for majority of the forested areas in Poland, the *S. bovinus* is a moderate accumulator of Hg. When compared with the established limits of Hg intake from foods, the consumption of *S. bovinus* does not pose toxicological concerns.
